# Supplementary material for: Recent Advances in Pancreatic Cancer: Novel Prognostic Biomarkers and Targeted Therapy—A Review of the Literature
Source: Biomolecules. 2021 Oct 6;11(10):1469. doi: 10.3390/biom11101469 (PMC8533343; doi:10.3390/biom11101469)
Supplement: Supplementary file 1 [file biomolecules-11-01469-s001.zip › biomolecules-1307479-supplementary.pdf]

Table S1: Overview of selected studies discussed in the text
If not otherwise specified, trials involve patients with locally advanced or metastatic PC

Drug/Target	Clinical trial number/name	Phase	Drug
**EGFR**			
erlotinib	NCT00026338[7]	3	Gemcitabine+erlotinib vs. gemcitabine
erlotinib (approved)	NCT00440167[8]	3	gemcitabine+erlotinib and capecitabine vs capecitabine+erlortinib and gemcitabine
cetuximab	NCT00075686[9]	3	gemcitabine+cetuximab vs. gemcitabine
erlotinib	Conko-005[10]	3	gemcitabine+erlotinib vs. gemcitabine (adjuvant)
**NTRK inhibitors (approved)**			
larotrectinib[11]	NCT02122913	1	Larotrectinib in tumors with NTRK fusion
	NCT02576431	2	Larotrectinib in tumors with NTRK fusion
	NCT02637687	1/2	Larotrectinib in children
Entrectinib[12]	NCT02097810	1/2a	Entrectinib in tumors with NTRK fusion
	NCT02568267	2	Entrectinib in tumors with NTRK fusion
**PARP inhibitors**			
Olaparib[13,14], **approved**	NCT01078662	2	Olaparib in all typs of advanced cancer with a germline BRCA1/2 mutation
	NCT02184195	3	Olaparib as maintance therapy for BRCA-mutated pancreatic cancer
Veliparib[15-17]	NCT01908478)	1	Veliparib plus gemcitabine and radiotherapy in locally advanced disease
	NCT01489865	1/2	Veliparib+FOLFOX in metastatic disease
	NCT01585805	2	Veliparib+ gemcitabine+cisplatin vs. gemcitabine+cisplatin with advanced pancreatic carcinoma
**K-Ras vaccination[18,19]**			
	(not available)		Adjuvant vaccination
	NCT03592888	1	Dentritic cell vaccine

Fibrosis			
**Hedgehog inhibitors**			
vismogedib[20]	NCT01064622	1b/2	Vismogedib+gemcitabine vs. gemcitabine
saridegib[21]	NCT01383538	1	FOLIRINOX+sarigedib
vismogedib[22]	NCT01088815.	2	vismogedib +gemcitabine/ nab-paclitaxel
**Hyoluronidase (Pegvorhyaluronidase Alfa)**	NCT02715804	3	Pegvorhyaluronidase Alfa (PEGPH20)+gemcitabine/nab-paclitaxel vs. gemcitabine/nab-paclitaxel
**TGF-beta (galunisertib)[6]**	NCT01373164	1b/2	Gemcitabine+galunisertib vs. gemcitabine
**Vitamin A receptor**	NCT03307148	2	ATRA+gemcitabinenab-paclitaxel
**VEGF**			
Axitinib[23]	NCT00471146.	3	Gemcitabine+axitinib vs. gemcitabine
Bevacizumab[24,25]	NCT00088894	3	Gemcitabine+bevacizumab vs. gemcitabine
	NCT00365144	3	Gemcitabine+erlotinib+bevacizumab vs. Gemcitabine+erlotinib
**PDL1/PD1**			
Pembrolizumab,[26] **approved**	NCT02628067	2	Pembrolizumab in dMMR/MSI-high tumors
Pembrolizumab (in combination)[27,28]	NCT02826486	2a	Pembrolizumab+ motixaforatide with chemotherapy
	NCT02620423<br>NCT03723915	1b<br>2	Pembolizumab+pelareorep
	NCT03634332	2	Pembrolizumab+PEGPH20
Spartalizumab	NCT04191421	1b/2	Spartalizumab+silutixmab
	NCT04581343	1	Spartalizumab+gemcitabine/nab-paclitaxel+canakinumab
Durvalumab[29]	NCT02558894	2	Durvalumab+tremilimumab vs durvalumab
**CLTA-4**			
Ipilimumab[30-34]	NCT04787991	2	Ipilimumab monotherapy
	NCT00836407	1	Ipilimumab+GVAX
	NCT02004262	2	GVAX+CRS-207
	NCT01473940	1	Ipilimumab+gemcitabine
	NCT01896869	2	Ipilimumab+ GVAX vs FOLFIRINOX as maintenance therapy

Nivolumab[35]	NCT02243371	2	Nivolumab+GVAX+CRS-207
CD40[36]	NCT00711191	1	Gemcitabine+CD40 monoclonal antibody CP-970-893
IL-10[37]	NCT02923921	3	Pegilodecakin+FOLOFX vs FOLOFX

1. Delle Cave D, Rizzo R, Sainz B, Gigli G, Del Mercato LL, Lonardo E. The Revolutionary Roads to Study Cell-Cell Interactions in 3D In Vitro Pancreatic Cancer Models. *Cancers (Basel)*. Feb 23 2021;13(4)doi:10.3390/cancers13040930
2. Cavo M, Delle Cave D, D'Amone E, Gigli G, Lonardo E, Del Mercato LL. A synergic approach to enhance long-term culture and manipulation of MiaPaCa-2 pancreatic cancer spheroids. *Sci Rep*. 06 23 2020;10(1):10192. doi:10.1038/s41598-020-66908-8
3. Hao Y, Baker D, Ten Dijke P. TGF-β-Mediated Epithelial-Mesenchymal Transition and Cancer Metastasis. *Int J Mol Sci*. Jun 05 2019;20(11)doi:10.3390/ijms20112767
4. Cave DD, Di Guida M, Costa V, et al. TGF-β1 secreted by pancreatic stellate cells promotes stemness and tumourigenicity in pancreatic cancer cells through L1CAM downregulation. *Oncogene*. 05 2020;39(21):4271-4285. doi:10.1038/s41388-020-1289-1
5. Norton J, Foster D, Chinta M, Titan A, Longaker M. Pancreatic Cancer Associated Fibroblasts (CAF): Under-Explored Target for Pancreatic Cancer Treatment. *Cancers (Basel)*. May 25 2020;12(5)doi:10.3390/cancers12051347
6. Melisi D, Garcia-Carbonero R, Macarulla T, et al. Galunisertib plus gemcitabine vs. gemcitabine for first-line treatment of patients with unresectable pancreatic cancer. *Br J Cancer*. 11 2018;119(10):1208-1214. doi:10.1038/s41416-018-0246-z
7. Moore MJ, Goldstein D, Hamm J, et al. Erlotinib plus gemcitabine compared with gemcitabine alone in patients with advanced pancreatic cancer: a phase III trial of the National Cancer Institute of Canada Clinical Trials Group. *J Clin Oncol*. May 2007;25(15):1960-6. doi:10.1200/JCO.2006.07.9525
8. Heinemann V, Vehling-Kaiser U, Waldschmidt D, et al. Gemcitabine plus erlotinib followed by capecitabine versus capecitabine plus erlotinib followed by gemcitabine in advanced pancreatic cancer: final results of a randomised phase 3 trial of the 'Arbeitsgemeinschaft Internistische Onkologie' (AIO-PK0104). *Gut*. May 2013;62(5):751-9. doi:10.1136/gutjnl-2012-302759
9. Philip PA, Benedetti J, Corless CL, et al. Phase III study comparing gemcitabine plus cetuximab versus gemcitabine in patients with advanced pancreatic adenocarcinoma: Southwest Oncology Group-directed intergroup trial S0205. *J Clin Oncol*. Aug 2010;28(22):3605-10. doi:10.1200/JCO.2009.25.7550
10. Sinn M, Bahra M, Liersch T, et al. CONKO-005: Adjuvant Chemotherapy With Gemcitabine Plus Erlotinib Versus Gemcitabine Alone in Patients After R0 Resection of Pancreatic Cancer: A Multicenter Randomized Phase III Trial. *J Clin Oncol*. Oct 2017;35(29):3330-3337. doi:10.1200/JCO.2017.72.6463
11. Hong DS, DuBois SG, Kummar S, et al. Larotrectinib in patients with TRK fusion-positive solid tumours: a pooled analysis of three phase 1/2 clinical trials. *Lancet Oncol*. 04 2020;21(4):531-540. doi:10.1016/S1470-2045(19)30856-3

12. Doebele RC, Drilon A, Paz-Ares L, et al. Entrectinib in patients with advanced or metastatic NTRK fusion-positive solid tumours: integrated analysis of three phase 1-2 trials. *Lancet Oncol*. 02 2020;21(2):271-282. doi:10.1016/S1470-2045(19)30691-6
13. Kaufman B, Shapira-Frommer R, Schmutzler RK, et al. Olaparib monotherapy in patients with advanced cancer and a germline BRCA1/2 mutation. *J Clin Oncol*. Jan 2015;33(3):244-50. doi:10.1200/JCO.2014.56.2728
14. Golan T, Hammel P, Reni M, et al. Maintenance Olaparib for Germline. *N Engl J Med*. 07 2019;381(4):317-327. doi:10.1056/NEJMoa1903387
15. Tuli R, Shiao SL, Nissen N, et al. A phase 1 study of veliparib, a PARP-1/2 inhibitor, with gemcitabine and radiotherapy in locally advanced pancreatic cancer. *EBioMedicine*. Feb 2019;40:375-381. doi:10.1016/j.ebiom.2018.12.060
16. Pishvaian MJ, Wang H, He AR, et al. A Phase I/II Study of Veliparib (ABT-888) in Combination with 5-Fluorouracil and Oxaliplatin in Patients with Metastatic Pancreatic Cancer. *Clin Cancer Res*. 10 2020;26(19):5092-5101. doi:10.1158/1078-0432.CCR-20-1301
17. O'Reilly EM, Lee JW, Zalupski M, et al. Randomized, Multicenter, Phase II Trial of Gemcitabine and Cisplatin With or Without Veliparib in Patients With Pancreas Adenocarcinoma and a Germline. *J Clin Oncol*. 05 2020;38(13):1378-1388. doi:10.1200/JCO.19.02931
18. Wedén S, Klemp M, Gladhaug IP, et al. Long-term follow-up of patients with resected pancreatic cancer following vaccination against mutant K-ras. *Int J Cancer*. Mar 2011;128(5):1120-8. doi:10.1002/ijc.25449
19. Abou-Alfa GK, Chapman PB, Feilchenfeldt J, et al. Targeting mutated K-ras in pancreatic adenocarcinoma using an adjuvant vaccine. *Am J Clin Oncol*. Jun 2011;34(3):321-5. doi:10.1097/COC.0b013e3181e84b1f
20. Catenacci DV, Junttila MR, Karrison T, et al. Randomized Phase Ib/II Study of Gemcitabine Plus Placebo or Vismodegib, a Hedgehog Pathway Inhibitor, in Patients With Metastatic Pancreatic Cancer. *J Clin Oncol*. Dec 2015;33(36):4284-92. doi:10.1200/JCO.2015.62.8719
21. Ko AH, LoConte N, Tempero MA, et al. A Phase I Study of FOLFIRINOX Plus IPI-926, a Hedgehog Pathway Inhibitor, for Advanced Pancreatic Adenocarcinoma. *Pancreas*. Mar 2016;45(3):370-5. doi:10.1097/MPA.0000000000000458
22. De Jesus-Acosta A, Sugar EA, O'Dwyer PJ, et al. Phase 2 study of vismodegib, a hedgehog inhibitor, combined with gemcitabine and nab-paclitaxel in patients with untreated metastatic pancreatic adenocarcinoma. *Br J Cancer*. 02 2020;122(4):498-505. doi:10.1038/s41416-019-0683-3
23. Kindler HL, Ioka T, Richel DJ, et al. Axitinib plus gemcitabine versus placebo plus gemcitabine in patients with advanced pancreatic adenocarcinoma: a double-blind randomised phase 3 study. *Lancet Oncol*. Mar 2011;12(3):256-62. doi:10.1016/S1470-2045(11)70004-3
24. Kindler HL, Niedzwiecki D, Hollis D, et al. Gemcitabine plus bevacizumab compared with gemcitabine plus placebo in patients with advanced pancreatic cancer: phase III trial of the Cancer and Leukemia Group B (CALGB 80303). *J Clin Oncol*. Aug 2010;28(22):3617-22. doi:10.1200/JCO.2010.28.1386
25. Van Cutsem E, Vervenne WL, Bennouna J, et al. Phase III trial of bevacizumab in combination with gemcitabine and erlotinib in patients with metastatic pancreatic cancer. *J Clin Oncol*. May 2009;27(13):2231-7. doi:10.1200/JCO.2008.20.0238

26. Marabelle A, Le DT, Ascierto PA, et al. Efficacy of Pembrolizumab in Patients With Noncolorectal High Microsatellite Instability/Mismatch Repair-Deficient Cancer: Results From the Phase II KEYNOTE-158 Study. *J Clin Oncol*. 01 2020;38(1):1-10. doi:10.1200/JCO.19.02105
27. Bockorny B, Semenisty V, Macarulla T, et al. BL-8040, a CXCR4 antagonist, in combination with pembrolizumab and chemotherapy for pancreatic cancer: the COMBAT trial. *Nat Med*. 06 2020;26(6):878-885. doi:10.1038/s41591-020-0880-x
28. Mahalingam D, Wilkinson GA, Eng KH, et al. Pembrolizumab in Combination with the Oncolytic Virus Pelareorep and Chemotherapy in Patients with Advanced Pancreatic Adenocarcinoma: A Phase Ib Study. *Clin Cancer Res*. 01 2020;26(1):71-81. doi:10.1158/1078-0432.CCR-19-2078
29. O'Reilly EM, Oh DY, Dhani N, et al. Durvalumab With or Without Tremelimumab for Patients With Metastatic Pancreatic Ductal Adenocarcinoma: A Phase 2 Randomized Clinical Trial. *JAMA Oncol*. Oct 2019;5(10):1431-1438. doi:10.1001/jamaoncol.2019.1588
30. Royal RE, Levy C, Turner K, et al. Phase 2 trial of single agent Ipilimumab (anti-CTLA-4) for locally advanced or metastatic pancreatic adenocarcinoma. *J Immunother*. Oct 2010;33(8):828-33. doi:10.1097/CJI.0b013e3181eec14c
31. Le DT, Lutz E, Uram JN, et al. Evaluation of ipilimumab in combination with allogeneic pancreatic tumor cells transfected with a GM-CSF gene in previously treated pancreatic cancer. *J Immunother*. Sep 2013;36(7):382-9. doi:10.1097/CJI.0b013e31829fb7a2
32. Le DT, Wang-Gillam A, Picozzi V, et al. Safety and survival with GVAX pancreas prime and Listeria Monocytogenes-expressing mesothelin (CRS-207) boost vaccines for metastatic pancreatic cancer. *J Clin Oncol*. Apr 2015;33(12):1325-33. doi:10.1200/JCO.2014.57.4244
33. Kamath SD, Kalyan A, Kircher S, et al. Ipilimumab and Gemcitabine for Advanced Pancreatic Cancer: A Phase Ib Study. *Oncologist*. 05 2020;25(5):e808-e815. doi:10.1634/theoncologist.2019-0473
34. Wu AA, Bever KM, Ho WJ, et al. A Phase II Study of Allogeneic GM-CSF-Transfected Pancreatic Tumor Vaccine (GVAX) with Ipilimumab as Maintenance Treatment for Metastatic Pancreatic Cancer. *Clin Cancer Res*. Oct 2020;26(19):5129-5139. doi:10.1158/1078-0432.CCR-20-1025
35. Tsujikawa T, Crocenzi T, Durham JN, et al. Evaluation of Cyclophosphamide/GVAX Pancreas Followed by Listeria-Mesothelin (CRS-207) with or without Nivolumab in Patients with Pancreatic Cancer. *Clin Cancer Res*. 07 2020;26(14):3578-3588. doi:10.1158/1078-0432.CCR-19-3978
36. Beatty GL, Torigian DA, Chiorean EG, et al. A phase I study of an agonist CD40 monoclonal antibody (CP-870,893) in combination with gemcitabine in patients with advanced pancreatic ductal adenocarcinoma. *Clin Cancer Res*. Nov 2013;19(22):6286-95. doi:10.1158/1078-0432.CCR-13-1320
37. Hecht JR, Lonardi S, Bendell J, et al. Randomized Phase III Study of FOLFOX Alone or With Pegilodecakin as Second-Line Therapy in Patients With Metastatic Pancreatic Cancer That Progressed After Gemcitabine (SEQUOIA). *J Clin Oncol*. Feb 2021:JCO2002232. doi:10.1200/JCO.20.02232

Table S2: Aberrant expression of micro- RNA inculding down and upregulation

**miRNA-**	**healthy vs pancreatic cancer**	**PanIn lesions**
21	up	
221	up	
424-5p	up	
27a	up	
4295	up	
205	up	
200	up	
210	up	
192	up	
18a	up	
124	down	
203	down	
150	down	
218	down	
22		aberrant
642		aberrant
10		aberrant
752		aberrant
12		aberrant
8885-5p		aberrant
196b		aberrant
155		aberrant
210		aberrant
1290	aberrant	
**miRNA- & Ca 19.9**		
16	aberrant	
196	aberrant	
27a-3p	aberrant	
**miRNA-**	**Expression**	**Survival**
21	up	worse
10b	up	worse
34a	down	worse
320c	up	better
200b	down	worse
200c	down	worse
let 7b	down	worse
let 7c	down	worse
let 7d	down	worse

let 7e	down	worse
33a	down	worse
15b	down	worse
17-5p	up	worse
155	up	worse
181c	up	worse
21	up	worse
203	up	worse
221	up	worse
320	up	worse
33a	down	better
138-5p	down	better
509-5p	down	better
1207	down	better
1243	down	better
7	down	worse
100	down	worse
124	down	worse
210	down	worse
200c	down	worse
205	down	worse
220b	down	worse
374b-5p	down	worse
497	down	worse
101-3p	down	better
15	down	better
203	down	better
205-5p	down	better
494	down	better
506	down	better
3656	down	better
